# Bite of a Rattlesnake Treated and Reported

**Published:** 1854-01

**Authors:** E. Stanley

**Affiliations:** Sandusky city, Ohio


### ART. IV.—*Bite of a Rattlesnake treated and reported.* By E. STANLEY, M. D., of Sandusky city, Ohio.

On the 9th of August, 1851, Patrick Burne, a young man, came to my office about 4 o'clock, P. M., seeking medical aid. I found him partially delirious; pulse very much excited ranging from 115 to 130; difficult and hurried respiration; skin hot and dry; eyes red and fiery; the hand, arm, and shoulder, swollen to a great degree; pain of the limb almost insupportable.

On making inquiry into the history of this case, I learned that the patient had been bitten about ten hours previously, and some forty miles from this city, by a rattlesnake.

This venomous reptile was concealed beneath a stick of timber which was intended for a tie on a railroad, and as the man was in the act of moving it, the wound was inflicted upon the index finger of the left hand, near the second joint.

Taking into consideration the length of time which had elapsed after the infliction of the wound, the general excited state of the system, and the poisonous appearance of the limb, I immediately ordered depletion by applying as many cups at one time to the arm and shoulder as would cover the surface, continuing this course for a number of hours without intermission, until about three quarts of blood was taken.

Prescribed poultices over wound, and ammonia and ether internally.

10th. Patient no better; delirious, pulse about the same, slept none, and suffered excruciating pain every moment during the night; slight nausea; no abatement of the swelling of the limb; arm, shoulder, and the upper portion of the left side, were thickly covered with small blisters, filled with a fluid of a yellowish color. In addition to former treatment, ordered whisky ad libitum, till the system was under its influence.

11th. Slight improvement; pulse about 100; swelling of the arm and shoulder a little diminished; still delirious, anxious and uneasy; very restless, dozed occasionally; skin hot and dry.

Same treatment with the addition of opium.

12th. Patient better; pulse less frequent; more quiet, and but little pain; slightly delirious; occasionally slept a few moments. Continued same prescription by adding to the whisky, capsicum, and administered it without regard to quantity, until the patient was fully under its influence. Ordered morphine to be given when symptons indicated it.

13th. A decided improvement; has passed the "crisis." Skin moist, pulse quite natural; enlargement of the arm and shoulder subsided; delirium had ceased, talked rationally, and is speedily recovering his usual health.

